# Hybridization and speciation in angiosperms: a role for pollinator shifts?

**DOI:** 10.1186/1741-7007-8-45

**Published:** 2010-04-21

**Authors:** Mark W Chase, Ovidiu Paun, Michael F Fay

**Affiliations:** 1 Jodrell Laboratory, Royal Botanic Gardens, Kew, Richmond, Surrey TW9 3DS, UK

## Abstract

The majority of convincingly documented cases of hybridization in angiosperms has involved genetic introgression between the parental species or formation of a hybrid species with increased ploidy; however, homoploid (diploid) hybridization may be just as common. Recent studies, including one in *BMC Evolutionary Biology*, show that pollinator shifts can play a role in both mechanisms of hybrid speciation.

See research article http://www.biomedcentral.com/1471-2148/10/103

## Hybridization as a mechanism of speciation in angiosperms

Species dynamics are often molded by gene flow between existing species due to interbreeding. Occasional interbreeding between species may have three main long-term outcomes: 1, genetic introgression into one or both of the hybridizing species, in some cases leading to assimilation of one species by the other; 2, formation of a new species without a change in ploidy (homoploid hybrid speciation); or 3, formation of a new species with a shift in ploidy (allopolyploidy). However, gene flow will most often have no long-term result, due to various isolation mechanisms that act to keep parental species apart either at the stage of hybrid formation or, later, at establishment of new hybrid derivatives [1]. Hybridization involving a change in ploidy has long been known to play a role in angiosperm evolution [2]. Successful establishment as independent species of such hybrid polyploid progeny easily succeeds because allopolyploids are immediately isolated from further interactions with parental taxa due to high levels of sterility in their progeny (due to uneven numbers of chromosome complements). Recently in an assessment of genetic distances between parents of hybrid species, it was shown that parental species of allopolyploids are significantly more genetically divergent than those of homoploid hybrid species [1]. Such a relationship seems mainly reinforced by fertility selection, resulting from the inability of homoploid hybrids to undergo normal meiosis if the parental chromosome complements have experienced too many rearrangements since they last shared a common ancestor. There appear to be genetic limits having to do with physical rearrangements of parental chromosomes (presumably caused by non-disjunction at meiosis) that make polyploidy more likely when the parental species are distantly related. Of course, documenting allopolyploidy is relatively easy and involves demonstrating that the hybrids have twice as many chromosomes (or more) as the parental species and that they retain fixed polymorphisms at protein-coding loci as documented by either protein studies, which were common in the 1980 and 1990s, or DNA sequencing/fingerprinting, which are the current methods of choice.

## Documenting homoploid speciation is difficult without large data sets

In contrast to allopolypoidy, documenting homoploid hybrid speciation is notoriously difficult. We wish to make clear that we are referring here to established hybrids that have become genetically isolated from their parents and are functioning as distinct evolutionary units, as *bona fide *species. Hybrid individuals form often [3], but in general are ephemeral, either dying off or being reabsorbed into one of their parental taxa, which leads to introgression between the parents but not increased species diversity. Introgression occurs frequently when the parental taxa have 'porous genomes', that is, genomes that are still arranged similarly enough for there to be few if any barriers to hybridization [4].

There are two principal ways to detect the presence of homoploid hybrid species: 1, looking for incongruence (genes that give different information about species relationships, that is, reveal different signals or evolutionary histories; shown by methods such as split decomposition [5]) in phylogenetic studies involving multiple independent loci; and 2, documenting linkage disequilibrium, markers closely linked being more likely to originate from the same parent and give the same ideas about relatedness than those further separated on the chromosome or on different chromosomes. The second method, which relies upon the assumption that hybrid speciation involves recombination [6] and homogenization of parental homologues, is by far the most powerful, but it also requires DNA sequences from a large number of loci and knowledge of their chromosome positions. The first method can often lead to confusing interpretations caused by other populational and stochastic processes (mainly persistent ancestral polymorphisms or gene duplications), and thus it too requires data from a large number of loci, becoming more powerful as the number of loci analyzed increases [7]. Therefore, for the great majority of plant groups, proving that a species has had a homoploid hybrid origin is difficult.

Thus far, fewer than 50 cases of homoploid hybridization have been documented in the angiosperms [1,8], but as more plant genomes are sequenced we are sure that this number will increase dramatically. Cases in which homoploid hybrid species have been documented thus far have often involved analyses of morphometric data or data that document the mixed morphological traits of the hybrids, with relatively small amounts of genetic data that fortuitously demonstrate the incongruence expected when loci have been derived from different parents. Congruence of morphological and genetic data compatible with homoploid hybridization is powerful enough to make convincing arguments, whereas evidence from either data type alone can easily be attributed to other populational and stochastic phenomena [7].

## Hybridization does not guarantee reproductive isolation

Once formed, hybrids, both allopolyploid and homoploid, face similar ecological challenges. As mentioned above, allopolyploids benefit from immediate genetic barriers to backcrossing onto their parents that do not exist for homoploid hybrids, facilitating their genetic isolation from the parental taxa. Homoploid hybrids, in contrast, must face further genetic interactions with their parents, which often result in their reabsorption into a parental gene pool and introgression in that parent. When they do become established, there are several potential reasons why they succeed: 1, they exhibit transgressive traits (novel mixtures of those exhibited by their parents [9]) that facilitate their existence in habitats in which neither of their parents can grow, conferring the isolation needed to prevent their reabsorption into the gene pool of one of their parents; or 2, their intermediacy in some morphological traits or ecological preferences enables them to exploit a novel niche, again delivering a degree of genetic isolation from both of their parents. The degree to which the latter mechanism is feasible has been widely debated. Often such hybrids can only exploit the ecological transition zone between those of their parents, which places them near one or both parents and which may be so limited in area that they cannot form viable populations. Such intermediates are unlikely to form viable species in their own right.

## Pollinator shifts as a mechanism of reproductive isolation hybrids

Hybridization leads to rapid genomic alterations, including chromosomal rearrangements and gene expression changes, some of which are mediated by transposable elements [10,11]. These genomic changes often result in novel phenotypes, some that are intermediate between parentals, some that represent novel combinations of parental features, and, finally, others that are extreme or transgressive compared to those of the parental species. Homoploids with flowers intermediate between their parents in morphology or that produce a complex mixture of floral fragrances from both parents are likely to be ineffective in attracting the pollinator of either parent, so there is likely to be strong selection to conform to the morphology of one or the other parent. This affects both homoploids and allopolyploids equally.

**Figure 1:**
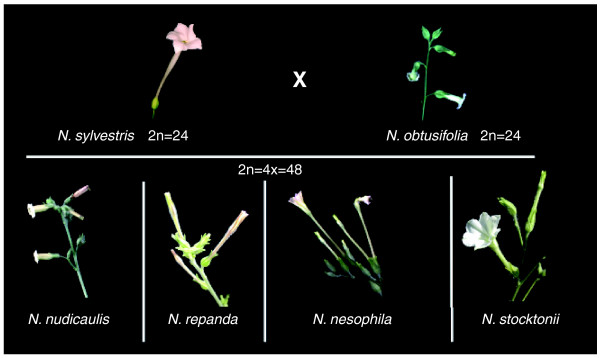
**Allopolyploid species of *Nicotiana *(Solanaceae) and their parental diploid species**. Note that the floral morphology of three of the polyploids, *N. repanda, N. nesophila *and *N. stocktonii *(allotetraploids, 2n = 48), is like that of *N. sylvestris *(a diploid, 2n = 24), adapted for nocturnal hawk-moth pollination, and the fourth, *N. nudicaulis*, is like that of the other parent, *N. obtusifolia *(2n = 24), adapted for bee pollination during the day. Photographs taken by Kar Yoong Lim.

In *Nicotiana*, for example, the four species in the allopolyploid *N. *section *Repanda *have flowers that attract long-tongued night-flying moths (three species) like *N. sylvestris *(the female parent) or short-tongued bees (one species) like *N. obtusifolia *(Figure 1). Although flowers of intermediate morphology and fragrance may not be well adapted to any existing pollinator, it is not always the case, particularly if there are highly specific interactions between plant and pollinator. Examples of this include when nectar spur length is highly correlated to tongue length of a particular pollinator [12] or sexual deception is operating as a pollinator attractant, as in the case of the orchid genus *Ophrys*. As shown in a recent paper in *BMC Evolutionary Biology *[13], pollinators of *O. arachnitiformis *and *O. lupercalis *are attracted by complex blends of fragrance compounds, and their hybrids produce a complete admixture of these, attracting a different species of bee (or other hymenopteran) than either of those pollinating the parental species. Thus, the hybrid is immediately reproductively isolated from its parents and, provided that it is fertile and has a suitable habitat, can become established as a species in its own right. This sort of circumstance is rare and would not be likely to occur in hybrids (regardless of ploidy) with less specific types of pollination syndromes because it is highly dependent on the well coordinated aspects of this relationship. Any shift in the fragrance bouquet can potentially mimic, by chance, the pheromones of another species of pollinating insect. Such a mechanism is not likely to occur in species in which nectar or pollen are attractants because then it is floral morphology alone that determines the sort of vector that can pollinate the flower.

Hybrid invasion of an alternative niche is likely to be successful if it parallels the reproductive isolation of its parents and thus subjects the hybrids to different selection pressures. *Penstemon clevelandii*, a homoploid hybrid of *P. centranthifolius *(red-flowered, hummingbird-pollinated) and *P. spectabilis *(lavender-flowered, wasp-pollinated) has established reproductive isolation by selection for a divergent pollination syndrome (magenta-flowered, bee- and hummingbird-pollinated) [14].

Overall, recent evidence has demonstrated the power of hybridization in creating new combinations of traits and genes responsible for niche divergence, both ecological and reproductive. As more examples of homoploid hybridization are identified, we predict that the frequency of successful niche novelties will also increase.
